# Possible roles of amyloids in malaria pathophysiology

**DOI:** 10.4155/fso.15.43

**Published:** 2015-09-01

**Authors:** Ernest Moles, Juan José Valle-Delgado, Patricia Urbán, Isabel G Azcárate, José M Bautista, Javier Selva, Gustavo Egea, Salvador Ventura, Xavier Fernàndez-Busquets

**Affiliations:** 1 Nanomalaria Group, Institute for Bioengineering of Catalonia (IBEC), Baldiri Reixac 10–12, ES-08028 Barcelona, Spain; 2 Barcelona Institute for Global Health (ISGlobal, Hospital Clínic-Universitat de Barcelona), Rosselló 149–153, ES-08036 Barcelona, Spain; 3 Department of Biochemistry & Molecular Biology IV, Universidad Complutense de Madrid, Ciudad Universitaria, ES-28040 Madrid, Spain; 4 Research Institute Hospital 12 de Octubre, Universidad Complutense de Madrid, Ciudad Universitaria, ES-28040 Madrid, Spain; 5 Department of Cell Biology, Immunology & Neurosciences, University of Barcelona School of Medicine & Institut d'Investigacions Biomèdiques August Pi i Sunyer (IDIBAPS), ES-08036 Barcelona, Spain; 6 Institut de Biotecnologia i Biomedicina & Departament de Bioquímica i Biologia Molecular, Universitat Autònoma de Barcelona, ES-08193 Bellaterra, Spain

**Keywords:** amyloids, intrinsically unstructured proteins, malaria, prions

## Abstract

The main therapeutic and prophylactic tools against malaria have been locked for more than a century in the classical approaches of using drugs targeting metabolic processes of the causing agent, the protist *Plasmodium* spp., and of designing vaccines against chosen antigens found on the parasite's surface. Given the extraordinary resources exhibited by *Plasmodium* to escape these traditional strategies, which have not been able to free humankind from the scourge of malaria despite much effort invested in them, new concepts have to be explored in order to advance toward eradication of the disease. In this context, amyloid-forming proteins and peptides found in the proteome of the pathogen should perhaps cease being regarded as mere anomalous molecules. Their likely functionality in the pathophysiology of *Plasmodium* calls for attention being paid to them as a possible Achilles’ heel of malaria. Here we will give an overview of *Plasmodium*-encoded amyloid-forming polypeptides as potential therapeutic targets and toxic elements, particularly in relation to cerebral malaria and the blood–brain barrier function. We will also discuss the recent finding that the genome of the parasite contains an astonishingly high proportion of prionogenic domains.

**Figure 1.**
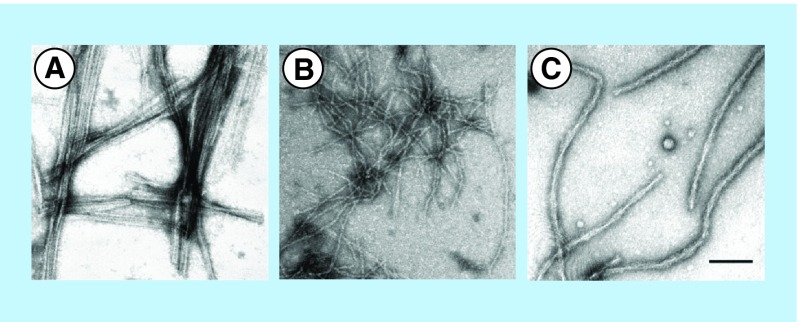
**Electron micrographs of the fibrils formed by (A) MSP2 8–15 peptide, (B) MSP2 1–25 peptide and (C) recombinant full-length FC27 MSP2.** Peptides and protein were incubated in PBS at 1 mg/ml. Samples were negatively stained and viewed using transmission electron microscopy. The scale bar represents 100 nm. Reproduced with permission from [6] © European Peptide Society and John Wiley & Sons, Ltd. (2007).

**Figure 2.**
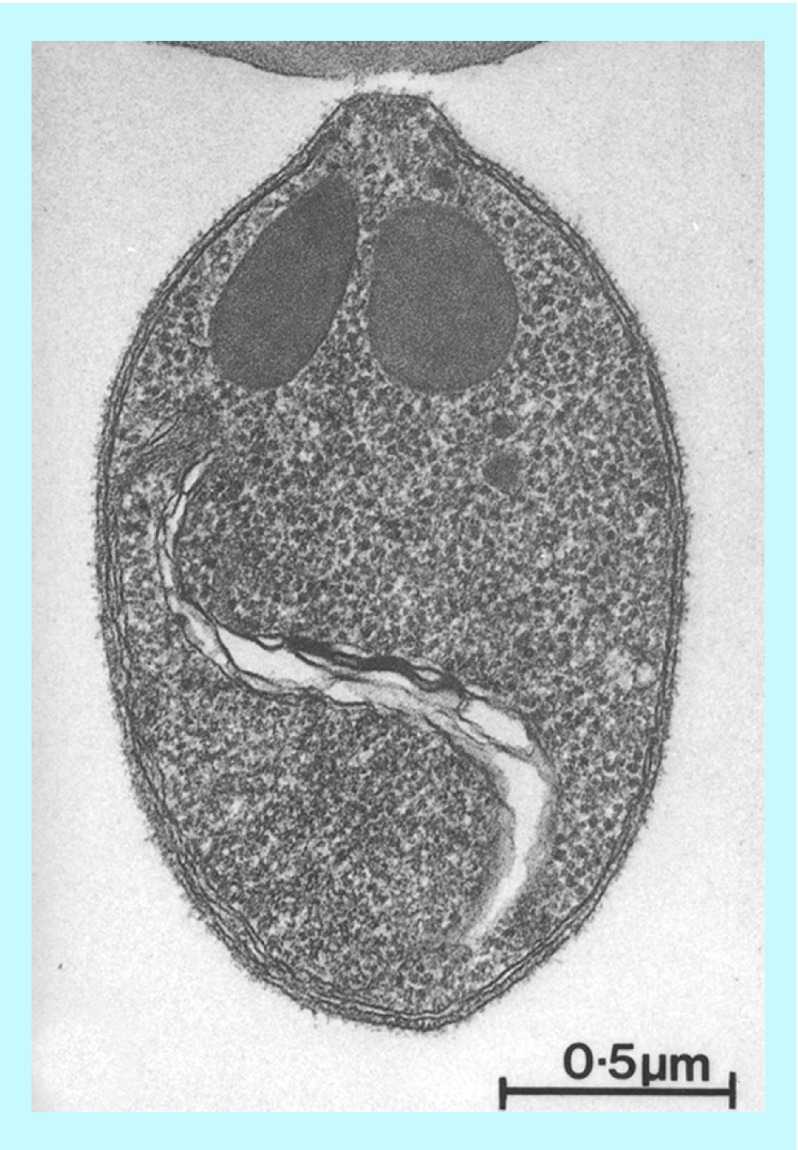
**Longitudinal section of a *Plasmodium knowlesi* merozoite showing the bristly appearance of the cell coat which covers the entire surface.** In this example, the parasite is attached apically to a red cell by a few long thread-like projections. Image reprinted with kind permission from [10]. Springer Science+Business Media B.V.

**Figure 3.**
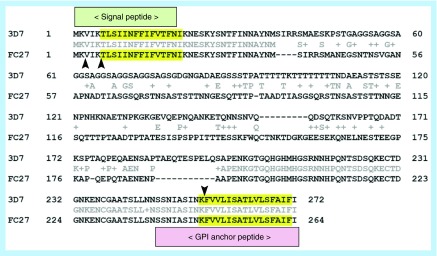
**Prediction of amyloid-forming sequences in the MSP2 isoforms 3D7 and FC27. Highlighted in yellow are the sequences with strong consensus prediction according to *FoldAmyloid, Tango, Waltz, Aggrescan* and *Zhang's method* algorithms.** The arrowheads indicate trypsin cleaving sites.

**Figure 4.**
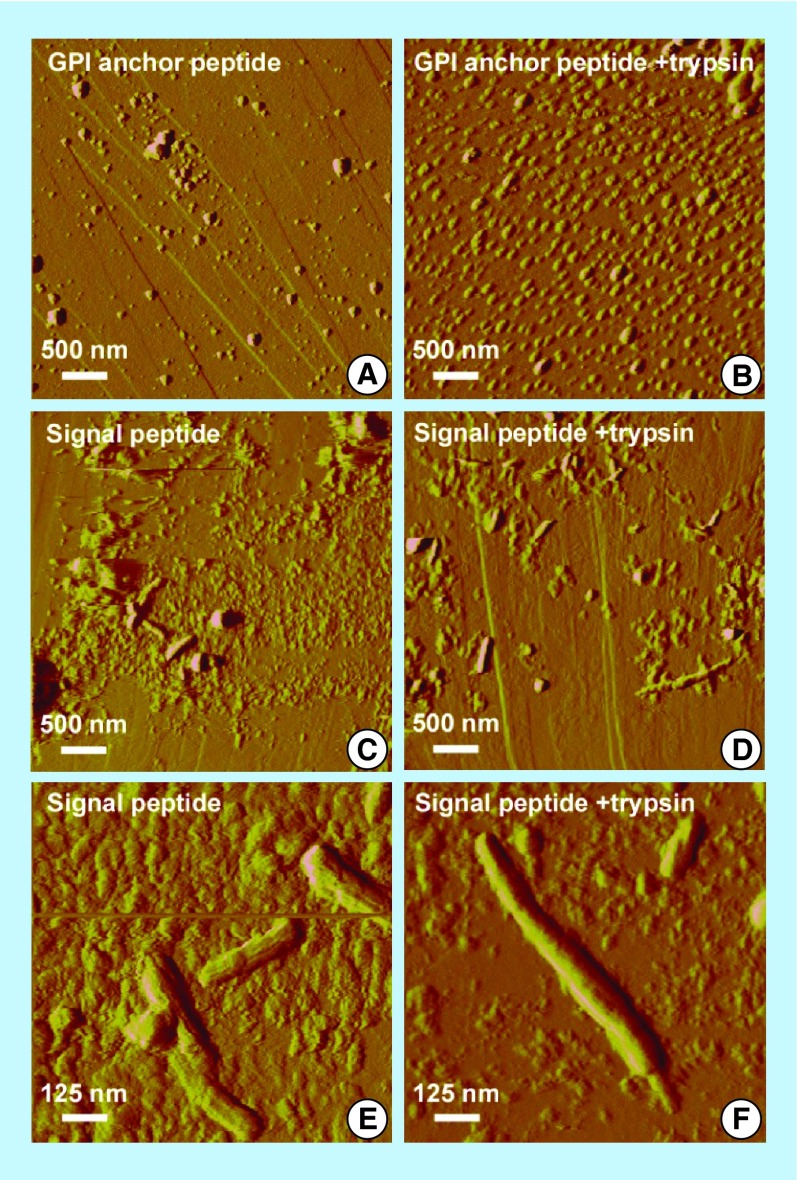
**Atomic force microscope images of aggregates of MSP2-derived peptides (digested or not with trypsin) formed after incubation in PBS at 37°C for 2–3 weeks.** The images were obtained using a Dimension 3100 atomic force microscope (Veeco Instruments, Inc., CA, USA) and NP-S probes (Veeco) with nominal spring constants of 0.06 N/m to scan the samples in tapping mode in liquid at 0.5–1.0 Hz scan rates. Highly oriented pyrolytic graphite (NT-MDT Co., Moscow, Russia) was used as a substrate.

**Figure 5.**
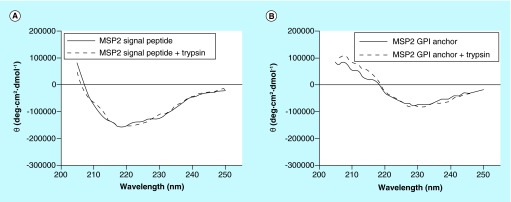
**Circular dichroism analysis of MSP2 (A) signal and (B) GPI anchor peptides, before and after trypsin digestion.**

**Figure 6.**
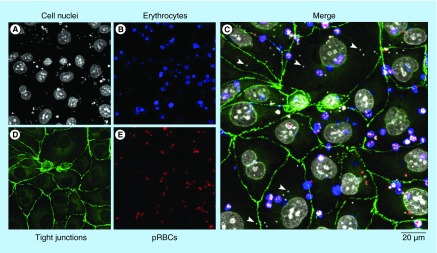
**Adhesion of parasitized red blood cells and of free *Plasmodium falciparum* parasites to an *in vitro* blood–brain barrier model.** HBMECs were initially expanded in a 75-cm^2^ flask precoated with fibronectin. *Plasmodium falciparum* strain E8B was grown *in vitro* in group B human erythrocytes using previously described conditions [26]. For the selection of cytoadherent parasites, pRBCs were passed over human umbilical vein endothelial cells (HUVECs) in two selection rounds. Late trophozoite- and schizont-containing pRBCs were selected in 70% Percoll and seeded at approximately 2 × 10^7^ pRBCs/cm^2^ on an HUVEC monolayer. The co-culture model of the BBB was adapted from a previously described protocol [27]. Briefly, rat astrocytes (RA) obtained from 2-day newborn rats were initially seeded at 60,000 cells/well onto a poly-D-lysine-coated plate. After 4 days, HBMECs were seeded at 16,500 cells/well onto collagen-coated 0.4 µm pore membrane inserts. This EC monolayer separated the system into an apical (bloodstream) and basolateral (brain tissue) compartments. The growth medium was changed every 3 days until cells reached confluence and tight junctions appeared. On the 9th HBMECs-RA co-culture day, pRBCs were purified in 70% Percoll, stained with wheat germ agglutinin-tetramethylrhodamine conjugate, and seeded at 1 × 10^6^ cells/well onto membrane inserts (apical compartment of the BBB model containing the EC layer). The system was co-cultured overnight at 37°C in a humidified atmosphere with 5% CO_2_ (protocol adapted from [28]). The day after, HBMECs and pRBCs were fixed and stained for the analysis by confocal microscopy of the tight junction marker protein zonula occludens-1 (ZO-1) [29]. Cell nuclei were stained with DAPI, and pRBCs were specifically detected with a monoclonal antibody against schizont-infected RBCs [26]. Arrowheads in the Merge panel indicate individual *P. falciparum* merozoites.

## Malaria pathophysiology

Malaria infection starts when a parasitized female *Anopheles* mosquito inoculates during a blood meal sporozoites of the malaria parasite, the apicomplexan protist *Plasmodium spp.*, which migrate through the skin into the circulation and then to the liver. In a few minutes, sporozoites invade hepatocytes, where they will transform into merozoites that enter the circulation to invade red blood cells (RBCs) [1]. Merozoites replicate asexually to produce daughter cells that infect new erythrocytes to perpetuate the blood-stage cycle. Some parasites eventually differentiate into sexual stages, micro- and macrogametocytes that are ingested by a mosquito from peripheral blood, and reach the insect's midgut where they develop into male and female gametes. Following fertilization, the zygote differentiates into an ookinete that moves through the midgut epithelium of the mosquito host and forms an oocyst from which sporozoites are released and migrate to the salivary glands to restart the cycle at the next bite. Because RBCs are unable to process and present antigens, the intraerythrocytic parasite remains invisible to the immune system until it starts modifying the parasitized RBCs (pRBCs) in order to meet its needs for membrane transport processes [1]. Even then, the proteins exported to the pRBC plasma membrane have a very high antigenic variation [2] which leads to waves of parasitemia and persistent infections despite antibody-mediated immune pressure.

## Amyloidogenic proteins & peptides in the malaria parasite

Sequence analysis has shown that apicomplexan parasite proteomes are enriched in intrinsically unstructured proteins (IUPs) [3], which contain large segments of disordered structure under physiological conditions. The IUP contents in mammalian-infecting *Plasmodium* species are particularly high, especially in the proteome of the *Plasmodium falciparum* sporozoite. Several *Plasmodium* IUPs have been examined as potential targets of interest for protective immune responses because repeats present in many of them are highly immunogenic showing strong reactivity with antibodies. However, antibodies raised against these repeats do not mediate protection in front of the parasite, probably because IUPs adopt more ordered structures when interacting with different ligands, and antibodies induced by such regions may recognize a variety of antigen conformers and thus react poorly with the antigen on the parasite surface. The structural plasticity of IUPs, which allows promiscuous binding interactions, may favor parasite survival both by inhibiting the generation of effective high affinity antibody responses and by facilitating the interactions with host molecules necessary for attachment to the vascular endothelium and for the invasion of host cells. Amyloidogenesis is undergone by a diverse group of evolutionarily unrelated proteins from different organisms, all sharing permanent or transiently disordered structural regions with propensity to form β-sheet aggregates in their complete or fragmented forms.

Among several merozoite surface proteins being assessed as potential components of a vaccine against *P. falciparum*, merozoite surface protein 2 (MSP2) is unusually hydrophilic and contains tandem sequence repeats characteristic of IUPs [4]. MSP2 is highly polymorphic, with conserved N- and C-terminal domains flanking a central variable region, and is anchored into the plasma membrane of the merozoite by a C-terminal glycosylphosphatidylinositol (GPI) moiety. MSP2 was one of three components in a combination vaccine that significantly reduced parasite densities in Papua New Guinean children living in an area where the transmission intensity of *P. falciparum* is high, and the MSP2 isoforms 3D7 and FC27 have been shown to elicit significant antibody responses in clinical studies [5]. Recombinant forms of MSP2 have been recently described to polymerize into amyloid-like fibrils *in vitro* [4]. The formation of MSP2 fibrils seems to be driven by cross-β aggregation of the N-terminal regions, as suggested by the observations that the protein contains there a proteinase K-resistant core, and that synthetic peptides from that region self-assemble *in vitro* into fibrils with amyloid-like properties [6] (Figure 1). The fibrillar aggregation of MSP2 would significantly increase the conformational order of the monomeric subunit with consequences for the protein's antigenicity, and the propensity of MSP2 to form fibrils in solution has been suggested to be an obstacle for its development as a malaria vaccine candidate [7].

Amyloid-like protein aggregates are associated with the surface of several microorganisms [8]. Functional amyloids have been found in bacteria, fungi and mammals with roles as diverse as biofilm formation, development of aerial structures, scaffolding, regulation of melanin synthesis, and activation of haemostatic factors, among others [9]. The *P. falciparum* merozoite surface bears a thick bristly coat, each bristle being a clump of thin (2–3 nm) filaments anchored at their bases to the plasma membrane, as in other species of *Plasmodium*, such as *P. knowlesi* [10] (Figure 2). Their removal by trypsin and papain enzymes points to a proteinaceous nature of those filaments.

Indirect evidence that MSP2 could form oligomers on the merozoite surface has been obtained from immunofluorescence experiments with monoclonal antibodies 6D8 and 11E1. 6D8, which preferentially recognized monomeric MSP2, failed to bind merozoites, in contrast to 11E1, which recognized both monomeric and polymeric MSP2 [4]. Amyloid-like protein aggregates on other microorganisms have roles in attachment and invasion of substrates. It will be of considerable interest to determine whether putative MSP2 amyloids on the surface of *P. falciparum* merozoites had similar functions, particularly given the reports of some forms of amyloid interacting with red blood cells [11,12]. However, the recent observation that MSP2 is carried into the host erythrocyte on the surface of the invading merozoite and then rapidly degraded [13] makes it unlikely that the mature protein has any toxic effects even in the case that it were found to form amyloids *in vivo*.

Somewhat surprisingly, analysis of the peptide sequence of MSP2 isoforms 3D7 and FC27 with the amyloid sequence-predicting algorithms [14] *FoldAmyloid, Tango, Waltz, Aggrescan* and *Zhang's method* indicated that the main amyloid-prone regions are actually found within the N-terminus signal peptide and the C-terminus GPI anchor signal peptide, which are not present on the processed mature protein (Figure 3). In Table 1 are shown the scores obtained using the *Waltz* algorithm, which is one of the most specific servers for the prediction of amyloid structure. Taking into account the values and also the peptide lengths (since the average score is per residue), we can possibly conclude that both MSP2 signal peptides are at least as amyloidogenic as the amyloid β peptide (Aβ). The N-terminus amyloidogenic region within the signal peptide is probably lost at the beginning of the protein transit through the endoplasmic reticulum (ER), whereas the C-terminus sequence within the GPI anchor signal peptide is excised later in the polypeptide processing. Because MSP2 is very abundant on the merozoite surface [15], these two peptides will likely be present at high concentrations within the ER, where they could start forming amyloid-like fibrils.

We performed *in vitro* assays to explore if both peptides formed amyloid-like structures in agreement with the theoretical predictions presented above. Two peptides derived from the MSP2 GPI anchor and signal sequences, SSNIASINKFVVLISATLVLSFAIFI-KKKK (Peptide Synthesis Unit, Scientific and Technological Centers of the University of Barcelona, Spain), and MKVIKTLSIINFFIFVTFNI-KKKK (Caslo Laboratory ApS, Lyngby, Denmark), both >95% purity according to chromatographic analysis, were incubated in phosphate buffered saline (PBS) at 37°C for 2–3 weeks. Since in both peptides the amyloidogenic region starts after or with a lysine (Figure 3), a second sample was incubated after trypsin digestion. Atomic force microscope (AFM) images on graphite (Figure 4) showed a propensity of the GPI anchor signal peptide to form globular structures, especially after trypsin cleavage, whereas the MSP2 signal peptide was observed to form amyloid-like fibrils up to several hundred nanometer in length. The presence of MSP2 on the merozoite surface suggests that its processing will occur during the terminal phase of the pRBC; the excised signal and GPI anchor peptides will likely have a very short time to be metabolized and thus might have the chance to form fibrils and oligomers that would be released in the blood when the pRBC bursts.

Circular dichroism analysis of the MSP2 signal peptide showed a minimum at 218 nm both before and after trypsin digestion (Figure 5), indicative of the presence of β-sheet structure characteristic of amyloid fibrils. The GPI anchor peptide did not exhibit a circular dichroism profile consistent with the existence of significant β-sheet structure, in agreement with AFM data indicating a reduced fibril formation propensity in comparison to the signal peptide.

As a first approximation to study the possible *in vivo* existence of amyloidogenic signal and GPI anchor MSP2 peptides, the plasma of malaria-infected persons was screened for the presence of antibodies against *in vitro* formed amyloid structures. ELISA analysis of the aggregated forms of MSP2 signal and GPI anchor peptides was performed using 71 sera samples from several sources as follows. Sixty sera were obtained from patients collected at a high-transmission region in Ghana, that were classified by the level of *P. falciparum* specific IgGs as high (7 × 10^6^–2 × 10^6^ ng/ml; n = 20), medium (1.5 × 10^6^–0.3 × 10^6^ ng/ml; n = 20) and low immunoreactive (5 × 10^4^ ng/ml; n = 20). In each group, half of the samples were malaria positive by PCR. In addition, 11 sera from Equatorial Guinea patients with MSP1 and/or MSP9-specific IgGs were also examined. Malaria nonexposed control sera were used to determine the baseline signal; all serum samples were from blood donors that had provided informed consent and the procedure was approved by the clinical research ethical committee at the University Hospital of Getafe (Spain). The analysis of the recognition of the aggregated forms of MSP2 signal and anchor peptides by all sera did not show any values above the threshold in ELISA assays. Although one interpretation of this result is that such forms are not present *in vivo*, we cannot rule out the possibility that amyloidogenic peptide forms have low immunogenicity, as it has been described previously [7]. If amyloidogenesis reduces immunogenicity, *Plasmodium* might have evolved amyloid-forming regions in proteins that are at some time exposed to the plasma.

## Cerebral malaria & integrity of the blood–brain barrier

Pathogenesis in Alzheimer's disease (AD) is linked to the accumulation of the highly amyloidogenic self-associating Aβ, whose oligomers, which are intermediate species in the assembly of fibrils, are powerful neurotoxins and might therefore be the key effectors of cytotoxicity [16]. We decided that it would be worth exploring the possibility that the presence of soluble amyloids encoded by the *Plasmodium* genome were related to some aspect of malaria pathophysiology, such as cerebral malaria (CM), the most severe neurological complication of falciparum malaria. Gene expression analysis suggested that CM and AD share common mechanisms of pathogenesis, as indicated by the accumulation of Aβ in brains of CM-susceptible (CM-S) but not in CM-resistant (CM-R) mice [17], in agreement with former reports showing detection of the Aβ precursor protein in humans with CM [18]. This result is consistent with the downregulation in CM-S animals of genes whose products inhibit the production of Aβ [17]. The reelin pathway that inhibits Tau protein phosphorylation, proposed as a protective mechanism against AD [19], has been found to be overexpressed in CM-R mice together with other genes involved in neurogenesis, suggesting that CM-S mice are less efficient in repairing neuronal damage [17]. In addition, decreased ribosomal RNA levels and decreased rates for protein synthesis have been described in AD, whereas downregulation of several genes encoding ribosomal proteins has been found in CM-S mice [17].

Erythrocytes infected with mature stages of the malaria parasite bind to the endothelial cells in the capillaries of tissues in a phenomenon known as sequestration, which allows *Plasmodium* to replicate while evading splenic clearance [20]. pRBCs can also adhere to noninfected RBCs giving rise to rosettes, and they can form clumps through platelet-mediated binding to other pRBCs. These events, which may lead to occlusion of the microvasculature, are thought to play a major role in the fatal outcome of CM. Because parasites are largely confined to intravascular spaces without entering the brain parenchyma, one main question regarding the pathogenesis of cerebral malaria is how neuronal dysfunction is caused [21]. There is growing evidence that *Plasmodium*-induced sequestration of infected and uninfected erythrocytes changes blood–brain barrier (BBB) function [22]. Entry of solutes from the blood into the CNS is characterized by a restricted permeability and active transport mechanism through the BBB formed by tight junctions between adjacent cerebrovascular endothelial cells (ECs). Postmortem analysis of CM brains has shown widespread disruption of these tight junctions, particularly in vessels containing pRBCs, which could result in the exposure of sensitive perivascular neuronal cells to toxic plasma metabolites [23]. pRBC binding to receptors on cerebral endothelial cells might cause changes in the integrity of the BBB, making it more permeable to molecules released upon egression of *Plasmodium* merozoites from the pRBC, among which perhaps neurotoxic amyloids formed by MSP2 signal peptides. Amyloid structures have been described to bind glycosaminoglycans such as the chondroitin and heparan sulfates of cell-bound glycoproteins and proteoglycans, including those present on ECs of the BBB [24]. Erythrocytes infected by the *P. falciparum* E8B strain and free parasites showed significant adherence to confluent human brain microvascular endothelial cells (HBMECs) in an *in vitro* BBB model (Figure 6). Although in this particular experiment no obvious alteration of tight junctions was observed, other studies performed under different conditions indicated disruption of the BBB induced by the malaria parasite [25].

## Prionogenic domains encoded in the malaria parasite genome

MSP2 has been the first *Plasmodium*-encoded protein unequivocally related to amyloid structures of potential pathological importance in malaria. However, recent data strongly suggest that in the short term, we will witness an important increase in the number of works dealing with the role in *Plasmodium* cell biology of a particular type of amyloids. When amyloid fibrils grow and divide with high efficiency they can propagate, become infective and transmit aggregative folds and are then termed prions [30]. While prions identified in mammals act usually as pathogens, prions in lower eukaryotes can be beneficial to the host [31]. There are organisms in which prion-like self-assembly might play important functions, as can be inferred from the rather high number of prions in their genomes. The inspection of incomplete sequenced genomes of some members of the genus *Plasmodium* proved that they might encode abundant hydrophilic low-complexity protein fragments corresponding to species-specific, rapidly diverging regions that could form nonglobular domains helping the parasites to evade the host's immune response [3]. Analysis of the complete proteomes of various members of this genus suggested that most of these stretches may be prionogenic domains [32]. In most cases, the fraction of proteins bearing prion-forming domains is less than 1% of the size of the proteome, but in *P. falciparum* the proportion of putative prions rises to an astonishing 10%. One in four proteins in this organism contains asparagine repeats, whose functions are still unknown [33], but are found in all protein families in all developmental stages. The life cycle of *Plasmodium* forces the organism through drastic changes in its environmental temperature, including cyclical fever episodes that can last several hours and exceed 40°C. Because heat shock increases the propensity of proteins to misfold and form aggregates, if only a fraction of the abundant prion-like domains in the malaria parasite aggregates during heat shock, this would certainly lead to the pathogen's demise. However, *Plasmodium* is able to survive with one of the most aggregation-prone proteomes in nature. This is due to the presence of specialized chaperones which prevent aggregation and are essential for parasite survival within RBCs [34]. Although prionogenic domains are potentially deleterious for the organism, their abundance in *Plasmodium* suggests that they might provide certain advantages, but the functional implications of such an amount of aggregation-prone proteins are still unclear. Identifying their so far unknown roles and mechanisms of action can provide new unexpected targets for future chemotherapeutic and prophylactic antimalarial approaches.

## Conclusion

The growing experimental evidence for the presence in the *Plasmodium* proteome of amyloidogenic peptides suggests that these entities have a physiological function in the life cycle of the pathogen. Functional amyloids might have roles in the adhesion of the parasite to its host cells during invasion, or participate in some of the pathological effects of malaria related, for example, to blood–brain barrier alteration or the triggering of immune responses. However, *Plasmodium*-derived amyloids could also provide new prophylactic or therapeutic possibilities if we are capable of harnessing them for future innovative antimalarial strategies.

**Table 1.** **Average scores obtained using the *Waltz* algorithm for the prediction of amyloid regions in MSP2 GPI anchor and signal peptides and in the two amyloidogenic stretches of the amyloid β peptide.**

**Peptide**	**Positions**	**Sequence**	**Average score per residue**
MSP2 GPI anchor	10–26	FVVLISATLVLSFAIFI	97.226048
MSP2 signal	6–19	TLSIINFFIFVTFNI	98.351648
Amyloid β	16–21	KLVFFA	97.993311
Amyloid β	37–42	GGVVIA	92.307692

**Executive summary**

At least one protein expressed by *Plasmodium*, MSP2, forms amyloid-like fibrils *in vitro*.
Amyloid-like structures formed by certain signal peptides of *Plasmodium* proteins might be released in the plasma.
Cerebral malaria and amyloid-related Alzheimer's disease share common mechanisms of pathogenesis.
*Plasmodium*-infected erythrocytes show adhesion to the blood–brain barrier according to *in vitro* assays.
The genome of *Plasmodium* encodes one of the highest proportions of putative prion sequences.
